# Synthesis and Biodegradation Test of a New Polyether Polyurethane Foam Produced from PEG 400, _L_-Lysine Ethyl Ester Diisocyanate (L-LDI) and Bis-hydroxymethyl Furan (BHMF)

**DOI:** 10.3390/toxics11080698

**Published:** 2023-08-13

**Authors:** Fabrizio Olivito, Pravin Jagdale, Goldie Oza

**Affiliations:** 1 Department of Chemistry and Chemical Technologies, University of Calabria, Via P. Bucci, Cubo 12C, 87036 Cosenza, Italy; 2 Circular Carbon GmbH, Europaring 4, 94315 Straubing, Germany; pravin.jagdale@circular-carbon.com; 3 Centro de Investigación y Desarrollo Tecnológico en Electroquímica, Pedro Escobedo 76703, Mexico; goza@cideteq.mx

**Keywords:** polyurethanes, renewable chemistry, degradation, enzyme, recycle

## Abstract

In this paper we produced a bio-based polyether-polyurethane foam PU1 through the prepolymer method. The prepolymer was obtained by the reaction of PEG 400 with _L_-Lysine ethyl ester diisocyanate (L-LDI). The freshly prepared prepolymer was extended with 2,5-bis(hydroxymethyl)furan (BHMF) to produce the final polyurethane. The renewable chemical BHMF was produced through the chemical reduction of HMF by sodium borohydride. HMF was produced by a previously reported procedure from fructose using choline chloride and ytterbium triflate. To evaluate the degradation rate of the foam PU1, we tested the chemical stability by soaking it in a 10% sodium hydroxide solution. The weight loss was only 12% after 30 days. After that, we proved that enzymatic hydrolysis after 30 days using cholesterol esterase was more favoured than hydrolysis with NaOH, with a weight loss of 24%, probably due to the hydrophobic character of the PU1 and a better adhesion of the enzyme on the surface with respect to water. BHMF was proved to be of crucial importance for the enzymatic degradation assay at 37 °C in phosphate buffer solution, because it represents the breaking point inside the polyurethane chain. Soil burial degradation test was monitored for three months to evaluate whether the joint activity of sunlight, climate changes and microorganisms, including bacteria and fungi, could further increase the biodegradation. The unexpected weight loss after soil burial degradation test was 45% after three months. This paper highlights the potential of using sustainable resources to produce new biodegradable materials.

## 1. Introduction

Biodegradable polymers based on renewable chemicals are becoming a concrete alternative to the materials produced using finite petroleum-derived sources [1,2,3,4]. The availability of agro-food waste and natural resources plays an important role, but from the environmental point of view, growing attention is paid to the use of green chemistry technologies [5,6,7]. The annual whole plastic production is estimated around 300 million tons/year [8], in which synthetic consumables derived from petrochemical hydrocarbon such as polyurethane (PU), polyethylene (PE), polyethylene terephthalate (PET), polystyrene (PS), polyvinyl chloride (PVC) and many others are still the most commonly produced [9,10]. Compared to global production, 18% of all plastic produced is recycled while 24% is incinerated. The remainder ends up in landfills or in the natural environment [11]. The degradation under natural conditions is a complex mechanism related to chemical, physical or biological events [12,13]. Despite the complexity of the topic, thanks to the efforts of industrial and academic research, promising progress has been made [14,15]. The biological degradation of polyethylene terephtalate is one of the recent innovations that have opened the views to promising future studies in this area [16]. Different biodegradable materials have been introduced in the past years [17]. Aliphatic esters such as polyhydroxyalkanoate (PHA) and polylactic acid (PLA) have been recognized as valuable alternatives to petro-plastics [18,19]. In particular, polyhydroxyalkanoates (PHAs) are emerging as versatile building blocks for plastic production and they are fully biocompatible and biodegradable in many environments [20,21]. PLA is another useful candidate obtained from renewable feedstocks and with low production costs, but for example, it showed some limitations regarding the degradation in seawater [22,23]. Polyurethanes are a class of polymers with a surprising number of applications ranging from engineering to medicine and the food industry [24,25]. These materials can be produced depending on the starting reagents as rigid, flexible or elastomeric foams [26]. The global annual production is estimated over 15 million tons per year in 2016 [27]. Polyurethane foams are still mainly produced using petroleum-derived chemicals by the classical reaction between isocyanates and polyols [28,29]. The global market of polyol was USD 26.2 billion in 2019 [30]. Despite this, the bio-based polyol market is constantly expanding, due to the awareness of the continuous consumption of finite resources [31,32]. Polyether polyurethanes are commonly difficult to degrade because the ether bonds limit the accessibility of the microorganisms and the relative enzymes, as well as they are not hydrolysable under normal conditions [33,34]. In this study, we used a mild and eco-friendly methodology to produce the renewable hydroxymethyl furfural (HMF) from fructose, in a biphasic system using choline chloride and ytterbium triflate [35]. The derivative 2,5-bis(hydroxymethyl)furan (BHMF) was obtained from HMF by a simple chemical reduction using sodium borohydride [36]. The final polyurethane PU1 was synthesized according to a two steps procedure in which the first step consists of the formation of the prepolymer while in the second step, BHMF is added for the elongation. In both stages, the catalyst used was sodium chloride [37,38]. Due to the growing concern about the toxic and carcinogenic effects of aromatic isocyanates which release dangerous diamines in the environment, we have decided to use a biodegradable and renewable diisocyanate which belongs to the amino acid-based diisocyanates, widely used over the past years in medicine [39,40]. Platform molecules derived from biomass, such as BHMF, HMF and FDCA together with other functionalized cellulosic materials are becoming versatile materials with ever-expanding applications [41,42,43]. Visible signs of degradation of polyurethanes are evident after 20–30 years and this mechanism strongly depends on the chemical composition [44,45]. In addition, polyether polyurethanes are reported to be more resistant to degradation with respect to polyester polyurethanes [46]. In this work, we investigated the short-term degradation of the bio-based polyether polyurethane foam PU1. We focused on chemical and enzymatic hydrolysis, and we obtained excellent results using cholesterol esterase. After the enzymatic hydrolytic degradation, we recovered BHMF through a simple extraction by an organic solvent, with the aim to recycle this monomer for further preparation of second-generation polymers [47] and to create a circular green process. In addition, thanks to the detection of this monomer we proposed a degradation mechanism. The soil burial degradation test, after three months, furnished even better results with respect to the enzyme treatment, probably due to the action of different natural agents such as microorganisms, sunlight, climate and others. This study opens new routes toward the use of renewable sources for the preparation of biodegradable materials. 

## 2. Materials and Methods

### 2.1. Materials 

Tetrahydrofuran (THF) was purchased from Carlo Erba (Milan, Italy) of analytical grade, and freshly distilled before use. 2-pentanone was purchased from Fisher scientific at a high purity grade and used without further purification. D-(-)-fructose was purchased from Sigma Aldrich with 99% purity and choline chloride was purchased from Thermo Fisher with 99% purity. Ytterbium triflate with 99.9% purity was purchased from Sigma Aldrich. Sodium borohydride was purchased from Carlo Erba (Milan, Italy) at 95% purity grade. Iodine was purchased from Carlo Erba (Milan, Itay) at the analytical grade. Polyethylene glycol (PEG) 400 was purchased from Thermo Fisher Scientific (Waltham, MA, USA) at 99% purity grade. L-Lysine ethyl ester diisocyanate (L-LDI) was purchased from Fisher Scientific, at 97% purity grade. Sodium chloride and sodium sulfate anhydrous were purchased from Sigma Aldrich (St. Louis, MO, USA) at analytical grade. Sodium hydroxide was purchased from VWR Chemicals 99% assay. Potassium phosphate monobasic was purchased from Sigma Aldrich in 99% assay. Potassium phosphate dibasic was purchased from Sigma Aldrich in 99% assay. IR spectra of the reagents are reported in the Supplementary Material (Sections S5 and S6). Cholesterol esterase isolated from bovine pancreas was purchased from Sigma Aldrich as lyophilized powder as ≥200 units/g protein. 

### 2.2. Methods

#### 2.2.1. Synthesis of HMF from d-Fructose

With a slight modification of the previously reported procedure, fructose (5 g) was mixed with choline chloride (1.5 molar equivalents) and ytterbium triflate (4% molar quantity with respect to fructose) in a 100 mL single neck round bottom flask. 2-pentanone (60 mL) was added. The system was connected with a condenser and stirred at 130 °C under reflux at atmospheric pressure for 2 h. After the defined time, the solution was decanted, dried with sodium sulfate anhydrous and filtered through a sintered glass filter, and the solvent was removed under a vacuum. The product in the form of yellowish oil with a yield of 90% was analyzed by GC-MS and HPLC with no trace of by-products [35]. 

#### 2.2.2. Synthesis of BHMF from HMF

A total of 3 g of HMF, obtained through the previously discussed method were dissolved in 60 mL of THF dry in a 100 mL one-neck round bottom flask. Sodium borohydride (NaBH_4_) in 25% weight with respect to HMF was added portion-wise. The open-flask reaction allowed the release of hydrogen produced during the reaction. After one hour, excess of sodium borohydride was quenched with aqueous HCl 1 N. The mixture was filtered through a sintered glass funnel. The solution was dried over sodium sulphate and filtered. The solvent was removed under vacuum to obtain a polyol in the form of a colourless oil, with a yield of 88%, and BHMF was identified as the main compound by HPLC and GC-MS [36].

#### 2.2.3. Prepolymer Synthesis

Water was used as a blowing agent at a weight percentage of 5.6% with respect to PEG 400. PEG 400, distilled water and NaCl as the catalyst (4% in weight with respect to the initial quantity of PEG 400) were added into a plastic container and the mixture was stirred using a mechanical apparatus. _L_-Lysine ethyl ester diisocyanate (L-LDI) was added at a molar excess of 2.5:1 with respect to PEG 400 and the mixture was vigorously stirred. The blend was warmed up to 70 °C for one hour until diisocyanate consumption. The prepolymer was obtained in the form of a colourless gel. The reaction was monitored by FT-IR spectroscopy with respect to the isocyanate signal due to the free isocyanate groups of the prepolymer [37].

#### 2.2.4. Polyurethane Synthesis Using BHMF for Chain Extension

BHMF was added in 30% weight with respect to the prepolymer and the mixture was vigorously and mechanically stirred for a few minutes in the same plastic container. After that, the same mixture was reversed in a steel mould and the system was closed under pressure at room temperature. The disappearance of the isocyanate signal was monitored by FT-IR spectroscopy. After 24 h, the polyurethane foam was obtained in the final form as a flexible white sponge and air-dried overnight [38].

#### 2.2.5. Hydrolytic Degradation

The hydrolysis was carried out at 37 °C using a 10% (*w*/*v*) solution of NaOH in distilled water. The foam was cut into small pieces of approximately 0.1 cm, after that a known quantity of 25 mg was put in 8 mL of alkali solution and stirred at 150 rpm for the period of time mentioned in the manuscript. After that, the foam was washed with distilled water and dried in an oven at 80 °C overnight. Weight loss is calculated with respect to the initial weight.

#### 2.2.6. Enzymatic Degradation

The phosphate buffer solution was prepared using 400 mL of distilled water in a 1 L flask, after that potassium phosphate monobasic (1.69 g) and potassium phosphate dibasic (10.10 g) were added and the solution was mixed. After adding 100 mL of distilled water the final pH of the solution was adjusted with NaOH or HCl (0.1N) to pH = 7. The polyurethane PU1 was cut into small pieces of approximately 0.1 cm and then a known quantity of 25 mg was put in 8 mL of the phosphate buffer solution together with 20 mg of the enzyme (dry powder) at 37 °C. Subsequently, the mixture was stirred at 150 rpm for the specified period of time, after which, the solution was filtered to a sintered glass funnel and washed with distilled water to remove the impurities. The remaining PU1 was dried in an oven at 80 °C overnight. The degradation was determined by weight loss because the polymer was totally insoluble in water and common organic solvents. BHMF was extracted from the aqueous solution by ethyl acetate. The organic phase was dried over magnesium sulphate and the solvent was removed under vacuum. The extracted organic phase was analysed by GC-MS. The aqueous phase was analysed by HPLC to detect any soluble by-products. 

#### 2.2.7. Soil Burial Degradation Test in the Garden

Polyurethane foam PU1 was cut into sheets of 1 × 1 cm^2^ with a thickness of 0.1 cm. The materials were buried in the soil garden to a depth of 20 cm. After the specified time, the material was removed from the soil, blown with compressed air and then washed with distilled water to remove the impurities. The foam was dried in an oven at 80 °C overnight. 

#### 2.2.8. Characterization

FT-IR was acquired by Nicolet Impact 410 FTIR Spectrometer (SpectraLab Scientific Inc., 38 McPherson St. Markham, ON, Canada L3R 3V6). Products (soluble in organic solvents) were analysed by GC-MS (Thermo Scientific, Waltham, MA, USA, TSQ 7000 equipped with a GC capillary column ROTI^®^Cap-1701 MS, 30 m, 0.25 mm, 0.25 µm, operating in the “split” mode, 1 mL min-1 flow of He as carrier gas) and quadrupole mass-detector. Products are soluble in the aqueous phase were analysed by Thermo Fisher, USA, UltiMate 3000 HPLC with isocratic pump and UV-vis detector. The reversed-phase analytical column used was a C18 ODS-A reversed-phase HPLC column, with dimensions of 120 A, 150 × 3 mm and 3 microns for particle size (Thomas Scientific, Chadds Ford, PA, USA). The wavelength of the reference was 360 nm with a peak width of 100, while the wavelength for the analysis was set to 385 nm with a peak width of 8 mm. The chosen mobile phase was methanol (49.99%) and acetonitrile (50%), in isocratic elution mode at a flow rate of 1 mL/min. The sample injection volume was 20 μL. SEM images were acquired with Hitachi High-Tech’s scanning electron microscopes SU3800, Tokyo, Japan. 

## 3. Results and Discussion

### 3.1. Synthesis of the Polyurethane PU1

**Figure 1:**
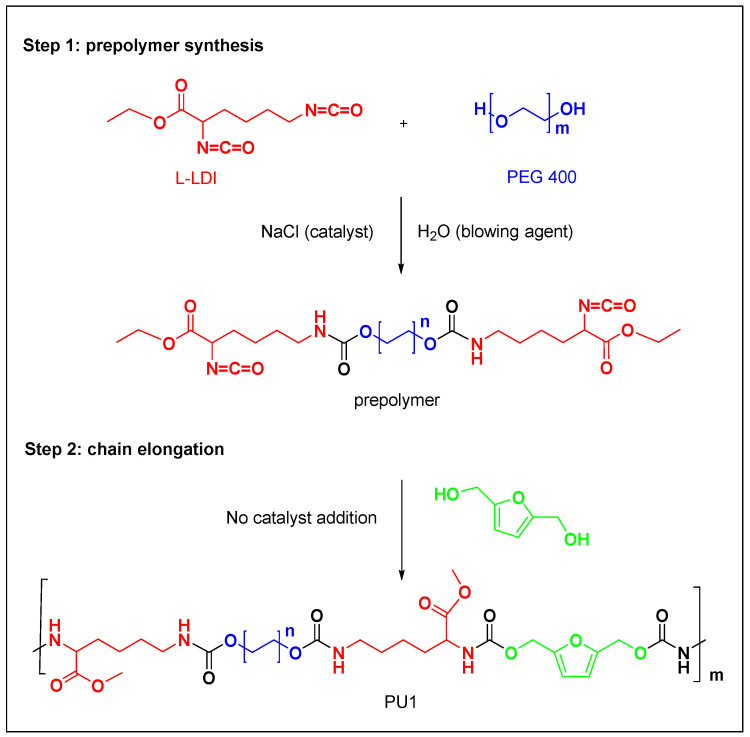
Synthesis of the polyurethane PU1.

We started with the preparation of the prepolymer following a previously reported procedure [37]. The reaction scheme with the prepolymer structure is reported in Figure 1. 

2,5 bis(hydroxymethyl)furan (BHMF) can be produced by a mild chemical reduction using sodium borohydride starting from HMF [36]. BHMF can be used as a chain extender in polyurethane synthesis [38]. The flexible foam PU1 was obtained after adding BHMF to the prepolymer and the reaction scheme with the main polyurethane chain was reported in the following Figure 1. 

#### FT-IR Characterization

**Figure 2:**
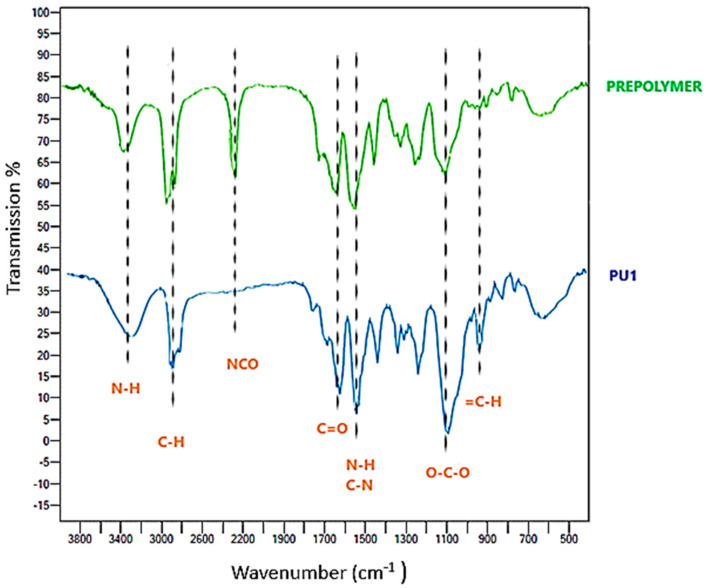
FT−IR spectra of the prepolymer and the final polyurethane PU1.

FT-IR spectra of the prepolymer and PU1 were reported in Figure 2. The absorption peaks of the prepolymer near 3400 cm^−^^1^, 2900 cm^−^^1^ and 2200 cm^−^^1^ are relative to N-H stretching vibrations, asymmetrical and symmetrical stretching vibrations of aliphatic C-H and NCO stretching vibration. The signal near 1700 cm^−^^1^ is due to carbonyl C=O stretching vibration related to the reagent L-LDI, which is probably shifted to lower frequencies, with respect to the normal value, due to interchain hydrogen bonds. The remaining signals near 1500 cm^−^^1^ and 1100 cm^−^^1^ and 950 cm^−^^1^ are relative to N-H bending vibrations and C-N stretching vibrations, O-C-O stretching frequency due to the reagent PEG 400 [38,39]. In the FT-IR spectra of PU1, the absorption band at 3400 cm^−^^1^ relative to N-H stretching vibration is more pronounced due to the longer chain of the final polyurethane with respect to the prepolymer and more urethane groups. Among the most significant signals, there is the complete disappearance of the isocyanate NCO stretching vibrations, confirming the completeness of the polymerization reaction, and the appearance at 950 cm^−^^1^ of the =C-H bending vibrations of furan, due to the introduction of BHMF as chain extender [48].

### 3.2. Biodegradability Tests

#### 3.2.1. Hydrolytic Degradation

**Figure 3:**
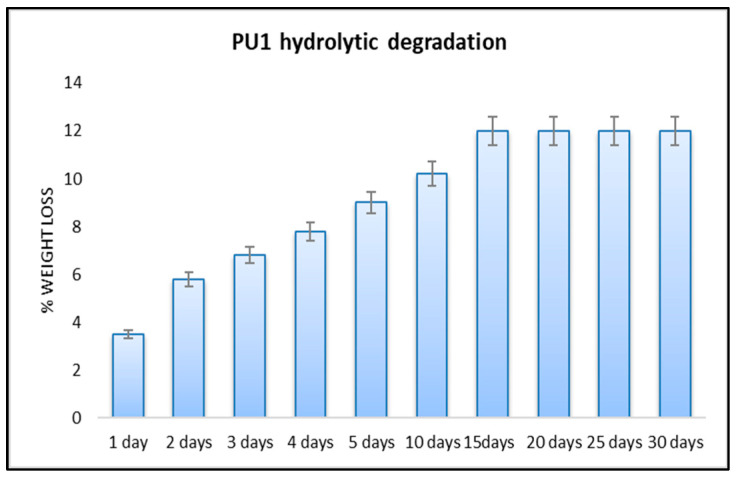
Weight loss of polyurethane PU1 after degradation in 10% NaOH solution.

We performed a non-enzymatic hydrolytic degradation by measuring the weight loss of PU1 using a 10% NaOH solution. This type of hydrolysis is usually favoured by the increase of hydrophilicity of the polymer [49,50]. This type of polyether polyurethane foam is not hydrophilic material due to the reagents used and this was proved by the values reported in the following Figure 3. 

In the case of alkaline hydrolysis, the chemical characteristics of this insoluble polyurethane PU1 produced limited contact between the aqueous solution and the hydrophobic surface. In fact, the percentage of degradation increases up to 15 days to a value of 12%, which remains constant for up to 30 days. The degradation products in these low values were not detected by GC-MS or HPLC. All measurements were repeated in triplicate and the values were expressed as the mean of the values.

#### 3.2.2. Enzymatic Degradation

**Figure 4:**
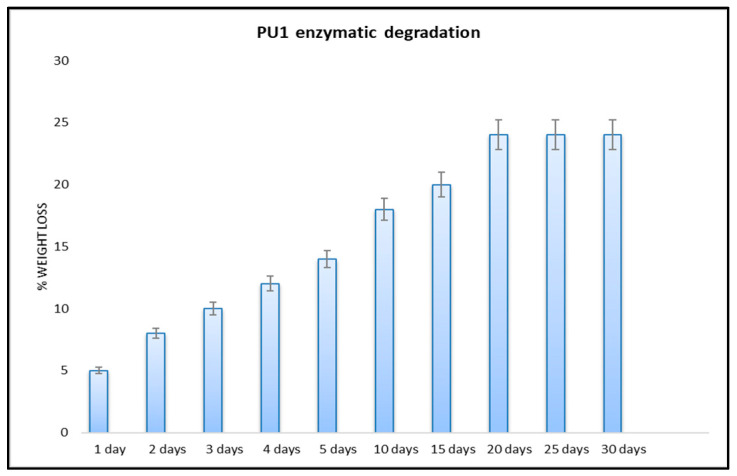
Weight loss of polyurethanes PU1 after degradation in cholesterol esterase (200 µg/mL in phosphate buffer with pH = 7).

Enzymatic degradation was evaluated by the measurement of the weight loss of the PU1 because we proved that it was completely insoluble in water or in the common organic solvents. Figure 4 shows the results relative to the enzymatic degradation using cholesterol cholesterase at different times in a phosphate buffer solution. 

For the enzymatic hydrolysis, the mechanism is expected to be divided into two stages, one of which is the enzyme adsorption on the hydrophobic surface and the second one is the hydrolysis of the hydrolysable sites [51]. The weight loss is fast during the first 20 days, then the value stays the same until 30 days. The weight lost by PU1 after four days is 12%, and after 10 days is 18%, confirming the fact that there is a positive and reactive enzyme activity until a constant value is reached. It is generally recognized that the enzymatic degradation activity preferentially occurs on the surface and decreases over time in the innermost structure [52,53]. These findings are novel because this is the first report of an enzymatic degradation related to a bio-based polyether polyurethane foam with supporting results [54]. All measurements were repeated in triplicate and the results expressed as the mean of the values.

**Figure 5:**
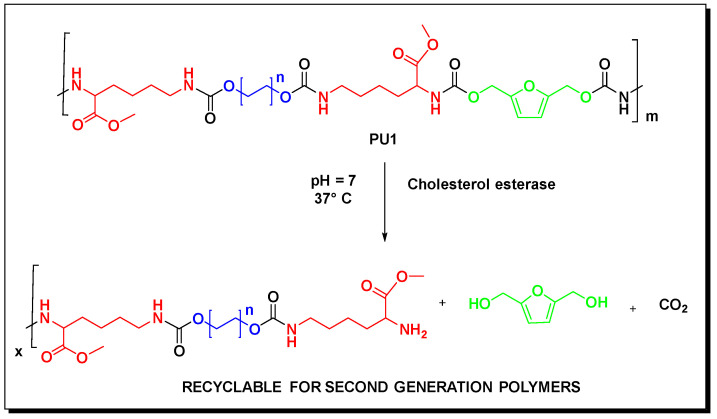
Supposed degradation mechanism of the polyurethane foam PU1 induced by cholesterol esterase in a water phosphate buffer at pH = 7.

We carried out a qualitative identification of the possible by-products by GC-MS and HPLC analysis (see ESI file). After the enzymatic hydrolysis test the aqueous solution was filtered and the organic phase was extracted several times using ethyl acetate, then analyzed by GC-MS. We have identified BHMF as the only molecule present in the organic phase (see the ESI file). We repeated the experiment without carrying out the extraction with the organic solvent and we analyzed the solution by HPLC. Also, in this case, using a standard, BHMF was the only identified by-product in the aqueous phase. From this experimental evidence, it was clear that BHMF represents the site of the enzymatic cleavage of the polymer chain of the polyether polyurethane foam. We reported the schematic representation of the supposed enzymatic hydrolysis mechanism in the following Figure 5. 

#### 3.2.3. Soil Burial Degradation

**Figure 6:**
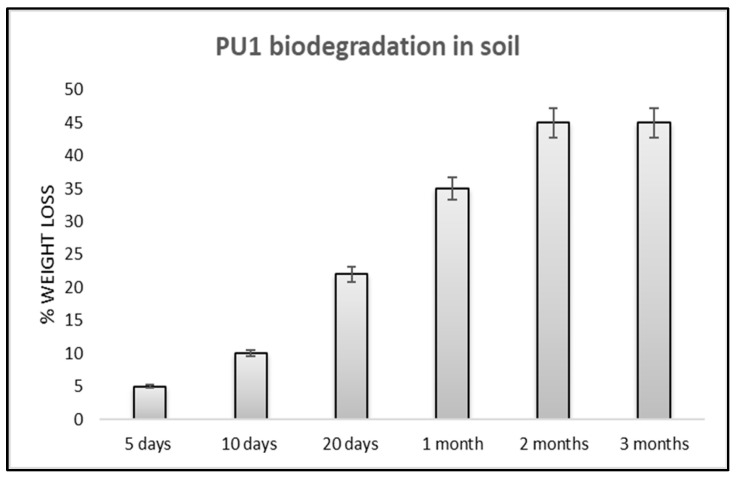
Weight loss of polyurethanes PU1 after soil-burial biodegradation.

To have a better understanding of the experimental results, we simulated a study of the biodegradability of PU1 directly in garden soil, at a pH of 7, an electrical conductivity of 0.8 (dS/m), the dry bulk density was 220 kg/mc and the total porosity was 82%. We buried the polyether polyurethane in the soil at a depth of 20 cm. The results relative to the weight loss of PU1 with respect to the time are reported in the following Figure 6. 

Different from enzymatic hydrolysis in which the material is treated with one type of enzyme, used in a relatively high concentration, several factors come into play in the case of biodegradation in soil, including climate changes, sunlight, water, bacteria, fungi and related extracellular enzymes produced by them [55,56]. From Figure 6, the degradation of PU1 is already high after one month, with a weight loss of 35%. The degradation increases after the first month, and at the control of two months, the weight loss was 45%. This initial degradation rate is related to the easy surface adhesion of the microorganisms on the hydrophobic surfaces of the material. After the second to the third month, the weight loss does not increase, confirming the fact that the internal part of the polyurethane is difficult to degrade in a short time. All measurements were repeated in triplicate and the values were expressed as the mean of the values.

#### 3.2.4. Properties of Polyurethane Foam before (PU1) and after (PU2) Biodegradation in Soil

##### FT-IR Spectra

**Figure 7:**
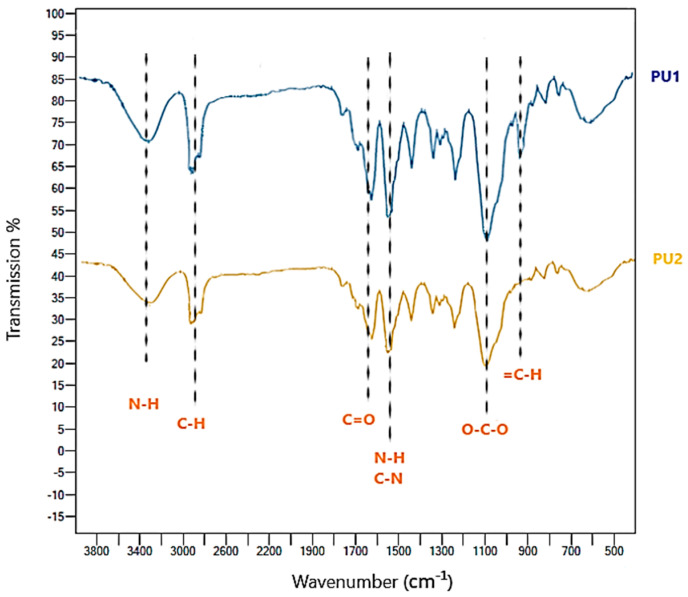
FT−IR spectra of the materials before (PU1) and after (PU2) the soil burial degradation test.

FT-IR spectra of the polyurethane foam before soil burial degradation (PU1) and after soil burial degradation (PU2) are reported in the following Figure 7.

The spectra of polyurethane PU2 after soil burial degradation shows the following characteristic signals of the synthetic polyurethane core more resistant to deterioration in analogy to the starting polyurethanes PU1: the IR signal near 3400 cm^−^^1^ is relative to N-H stretching vibrations, the signal near 3000 cm^−^^1^ is relative to symmetrical and asymmetrical stretching vibrations of aliphatic C-H, the signal near 1700 cm^−^^1^ probably corresponds to C=O stretching vibration. The broadband close to 1500 cm^−^^1^ n is likely to be relative to N-H bending vibrations and C-N stretching vibrations. The signal near 1100 cm^−^^1^ is relative to O-C-O stretching frequency [48,57]. The most important differences visible from the superposition of the IR spectra are two: firstly, the broadband near 3400 cm^−^^1^ is probably relative to N-H stretching vibrations of free amine groups and the urethane core has shifted in the biodegraded PU2 and is broader, this is probably due to the fact that the free NH_2_ groups formed after degradation are more available to take part in hydrogen bond networks with a relative shift to lower frequencies; secondly, we supposed during the discussion, that the breaking point of polyurethane PU1 employed in this study is 2,5 BHMF, and from the IR spectra in Figure 7 it is evident that for PU2 this signal in the region near 950 cm^−1^ relative to the =C-H bending vibrations of furan is absent respect to PU1 [58]. In conclusion, the hydrolysis of the site in which the renewable monomer 2,5 BHMF is present is also more susceptible to degradation after the soil burial degradation test. 

##### SEM Images

**Figure 8:**
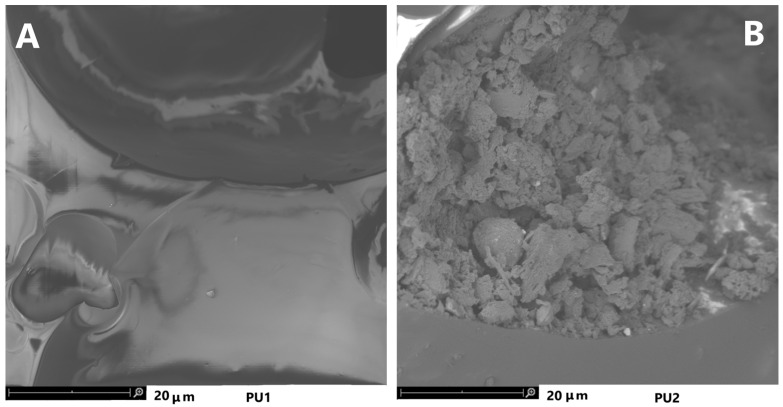
SEM images of the polyurethane before biodegradation in soil (**A**) and after three months of biodegradation in soil (**B**).

The last test that allowed us to obtain further confirmation of the degradation of the polyurethane PU1 was the morphological analysis by SEM Figure 8. 

In Figure 8A the surface of the newly synthesized PU1 polyurethane appears intact and uniform, while the SEM analysis of the PU2 (Figure 8B) which corresponds to the same polyurethane after 3 months of biodegradability tests in soil, shows evident signs of superficial decomposition, as we explained in the previous description, due to various natural agents.

## 4. Conclusions

In this work, we proved the surprisingly fast degradation of a flexible polyether polyurethane foam derived from renewable sources. We initially carried out a basic hydrolysis test with NaOH without obtaining satisfactory results. This was probably due to the hydrophobic nature of the polymer produced which does not have an efficient interaction with water and chemical substances dissolved in it. After that, we investigated the enzymatic hydrolysis with cholesterol esterase, and we obtained promising results with a weight loss of 24% after 20 days. We identified the breaking point of the polymer chain, and we found that BHMF is the center that undergoes hydrolytic attack by cholesterol esterase. The monomer BHMF together with the degraded polyurethane backbones can be recovered and reused to produce second-generation polymers. For a more realistic view of the investigation, we carried out the experiment on soil burial degradation in the garden. The obtained biodegradation was excellent, probably due to multiple factors like climate, microorganisms, secreted enzymes and others. The weight loss was 45% after three months. In this report, we investigated a promising degradation of a plastic-type material produced using renewable chemicals that open up an interesting future prospect in this sector.

## Data Availability

Not applicable.

## Supplementary Materials

The following supporting information can be downloaded at: https://www.mdpi.com/article/10.3390/toxics11080698/s1. Section S1: Reagents; Section S2: Determination of the isocyanate content of the prepolymer; Section S3: FT-IR spectra of PEG 400 and L-Lysine ethyl ester diisocyanate (L-LDI); Section S4: GC-MS and HPLC of HMF; Section S5: GC-MS and HPLC of BHMF; Section S6: GC-MS and HPLC of BHMF after enzymatic hydrolysis.
