# Supplementary material for: Synthesis and Biodegradation Test of a New Polyether Polyurethane Foam Produced from PEG 400, L-Lysine Ethyl Ester Diisocyanate (L-LDI) and Bis-hydroxymethyl Furan (BHMF)
Source: Toxics. 2023 Aug 13;11(8):698. doi: 10.3390/toxics11080698 (PMC10457969; doi:10.3390/toxics11080698)

Electronic supplementary information

Synthesis and biodegradation test of a new polyether polyurethane foam produced from PEG 400, L-Lysine ethyl ester diisocyanate (L-LDI) and bis-hydroxymethyl furan (BHMF)

Fabrizio Olivito [1,*], Pravin Jagdale [2] and Goldie Oza

[1] Department of Chemistry and Chemical Technologies, University of Calabria, Via P. Bucci, Cubo 12C, 87036 Rende, CS, Italy;
fabrizio.olivito@unical.it
[2] Circular carbon GMBH, Europaring 4, 94315 Straubing, Germany;
pravin.jagdale@circular-carbon.com
[3] Centro De Investigación Y Desarrollo Tecnológico En Electroquímica, Santiago de Querétaro, Mexico;
goza@cideteq.mx
* Correspondence: fabrizio.olivito@unical.it

Table of Contents

S1. Reagents **S3**

S2. Determination of the isocyanate content of the prepolymer **S4**

S3. FT-IR spectra of PEG 400 and L-Lysine ethyl ester diisocyanate (L-LDI) **S5**

S4. GC-MS and HPLC of HMF **S7**

S5. GC-MS and HPLC of BHMF **S8**

S6. GC-MS and HPLC of BHMF after enzymatic hydrolysis **S9**

S1. Reagents

Polyethylene glycol PEG 400 (Fisher Scientific)
Molecular formula: $(C_2H_4O)n$
Appearance (Color): Clear colorless
Form: Viscous liquid
Identification (FTIR): Conforms
Molecular Weight: 62.07 (g/mol)

L-Lysine ethyl ester diisocyanate (Fisher scientific)
Molecular formula: $C_{10}H_{14}N_2O_4$
Appearance (Color): Clear colorless
Form: liquid
Identification (FTIR): Conforms
Molecular Weight: 226.232 g/mol

S2. Determination of the Isocyanate content of the prepolymer

3-5 g of the prepolymer were dissolved in 50 ml of di-n-butylamine solution 0.2 N. 6 drops of bromophenol blue indicator were added. HCl 0.5 N was added until the colour of the solution turned to a yellow-green endpoint. The same procedure is carried out on the blank. The NCO % was calculated using the following formula:

$$\% NCO = \frac{4202\ (\mathrm{N})(\mathrm{B} - \mathrm{A})}{(sample\ weight)1000}$$

B = blank titer

A = sample titer

N = normality of the hydrochloric acid

*The prepolymer produced using the optimized reaction condition, present a NCO % of 15%, in accordance with the conventionally percentage reported industrially.

S3. FT-IR spectrum of PEG 400

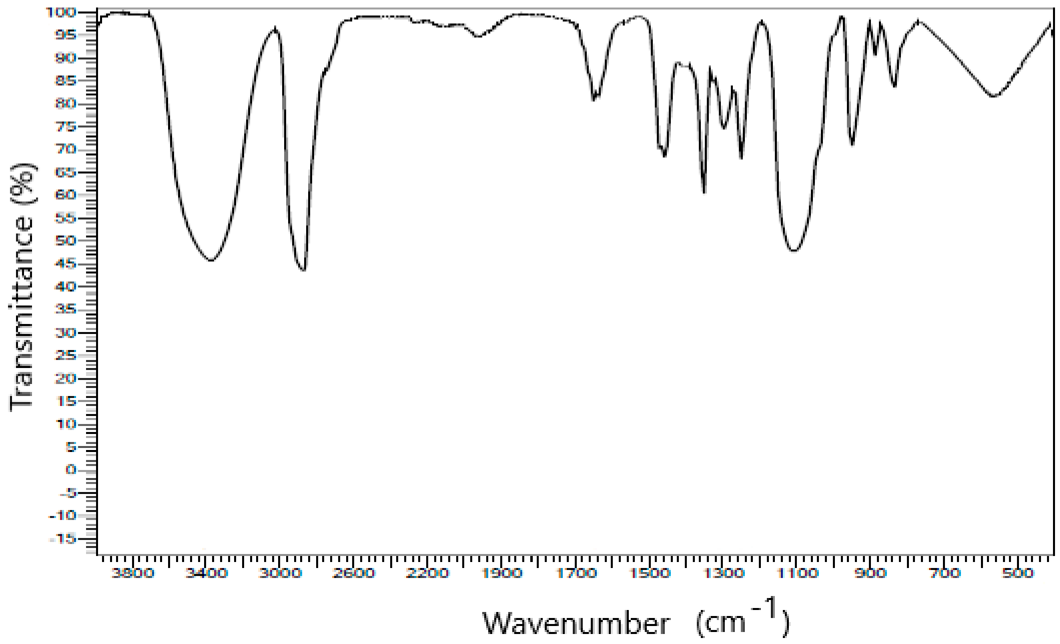

Functional group	Absorption peak (cm^{-1})
O-H	3379
Aliphatic C-H	2870
C-O-C and C-O-H	1103
Bending ethers	915-650

FT-IR spectrum of L-Lysine ethyl ester diisocyanate (L-LDI)

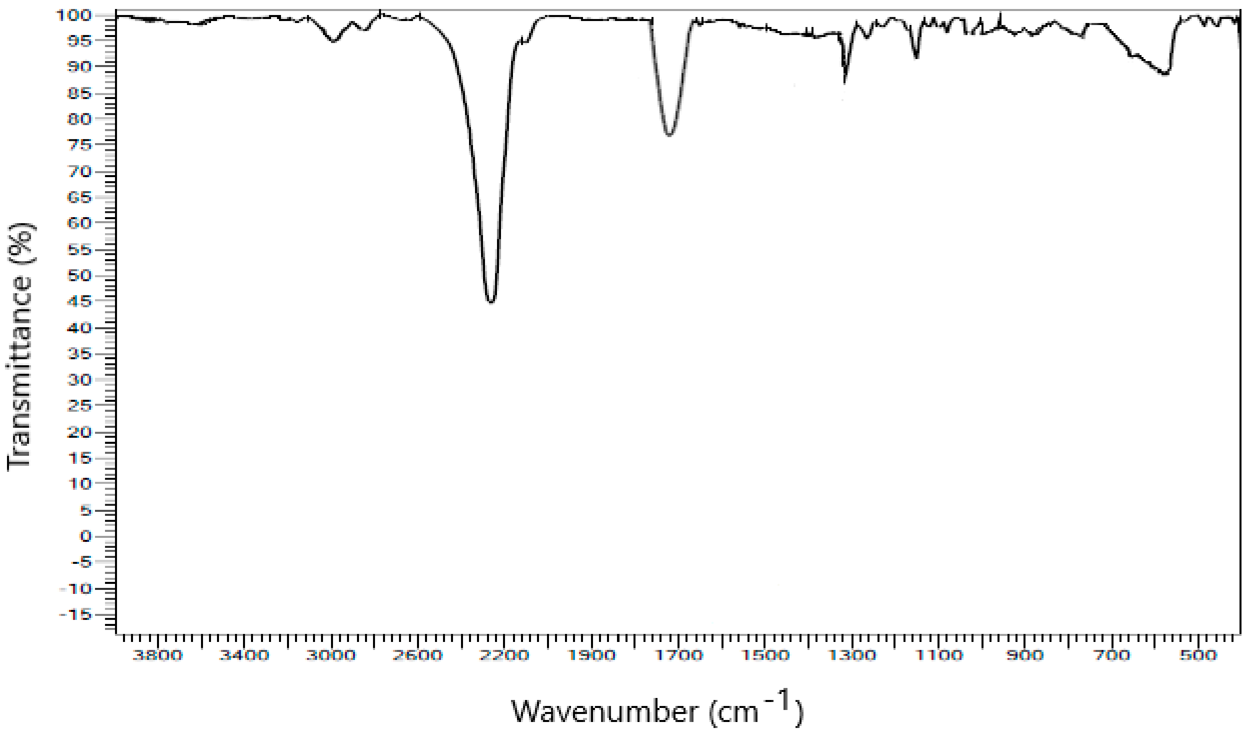

Functional group	Absorption peak (cm^{-1})
C-H stretching	2915
N=C=O stretching	2240
C=0	1740

S4. GC-MS and HPLC of HMF

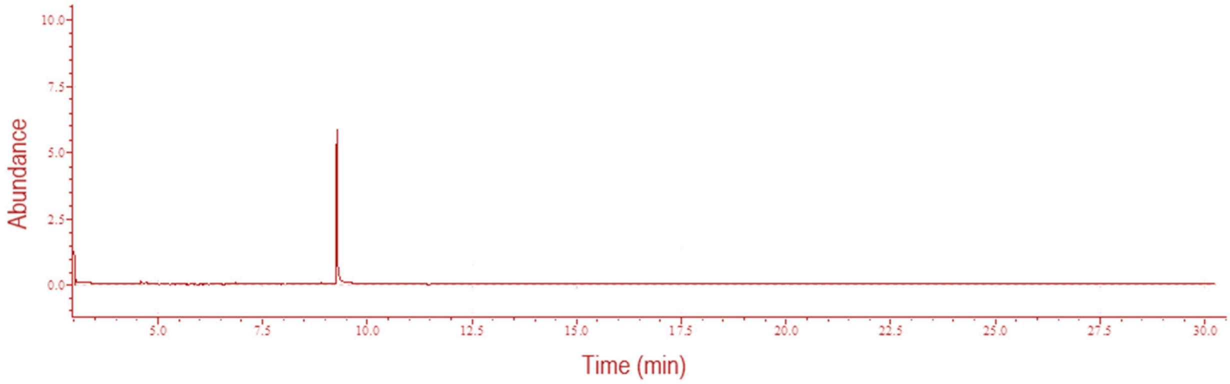

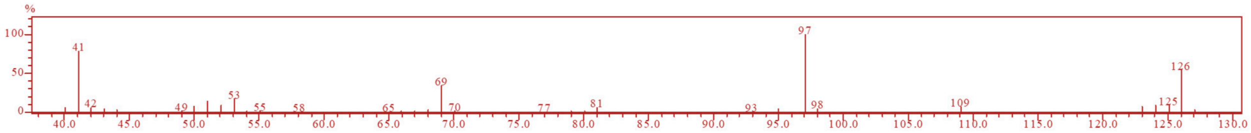

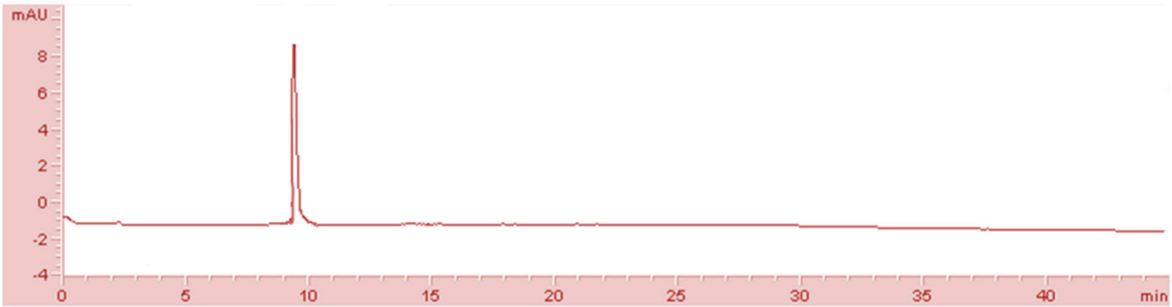

r.t. = 9.612

S5. GC-MS and HPLC of BHMF

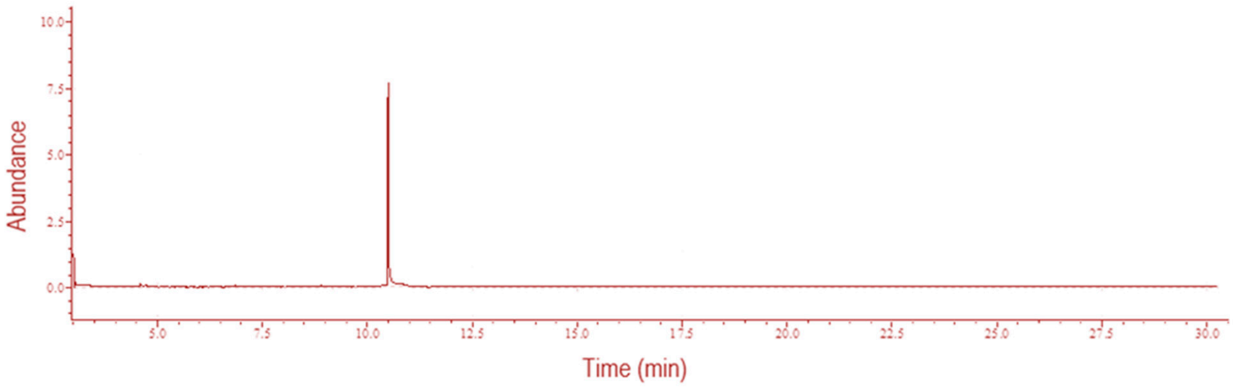

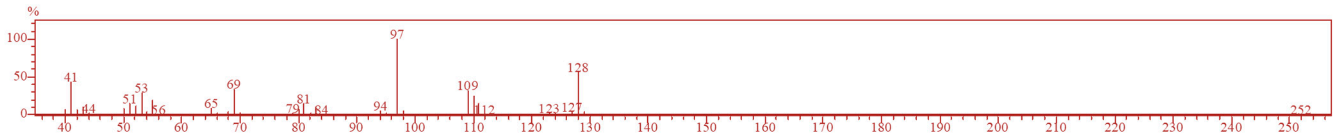

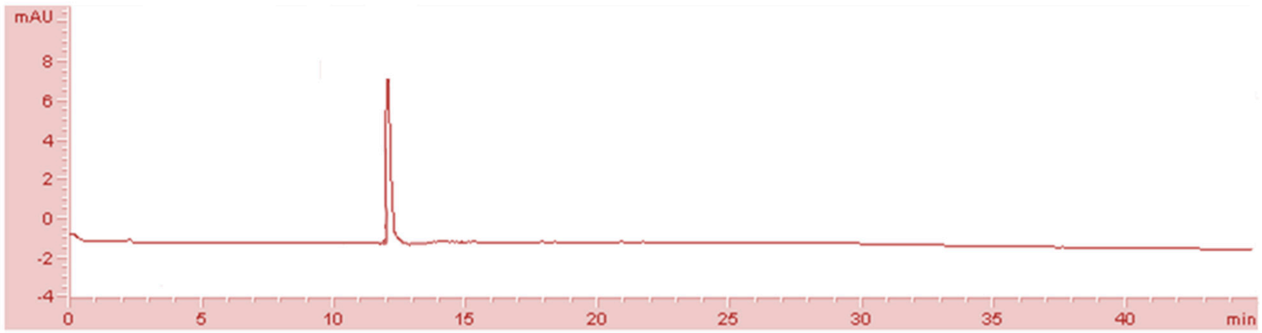

S6. GC-MS and HPLC of BHMF recovered after enzymatic hydrolysis

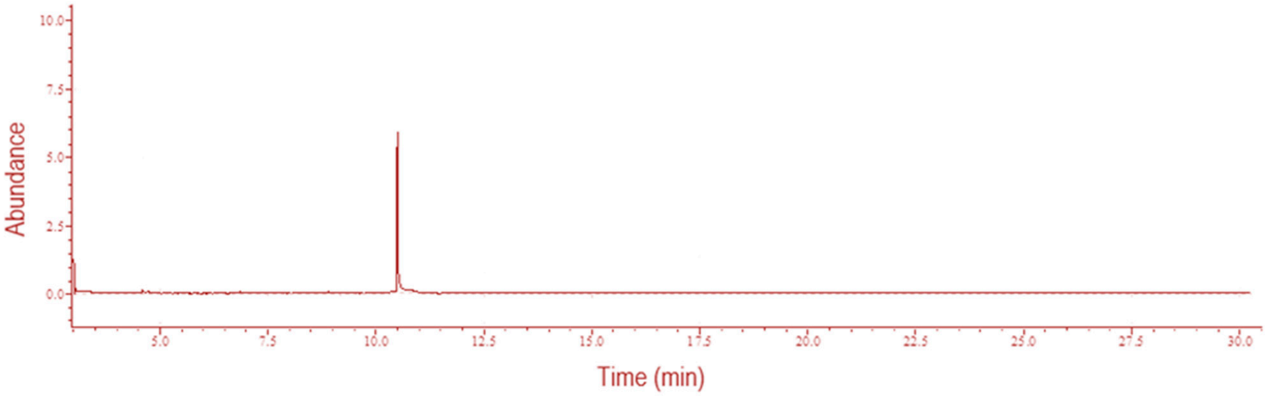

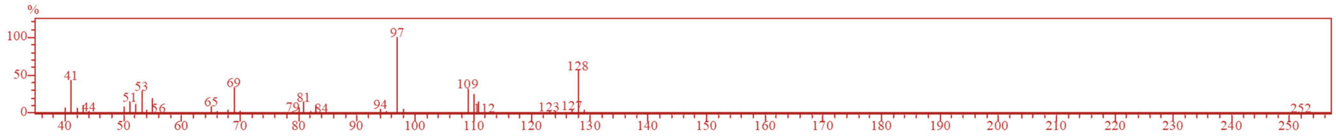

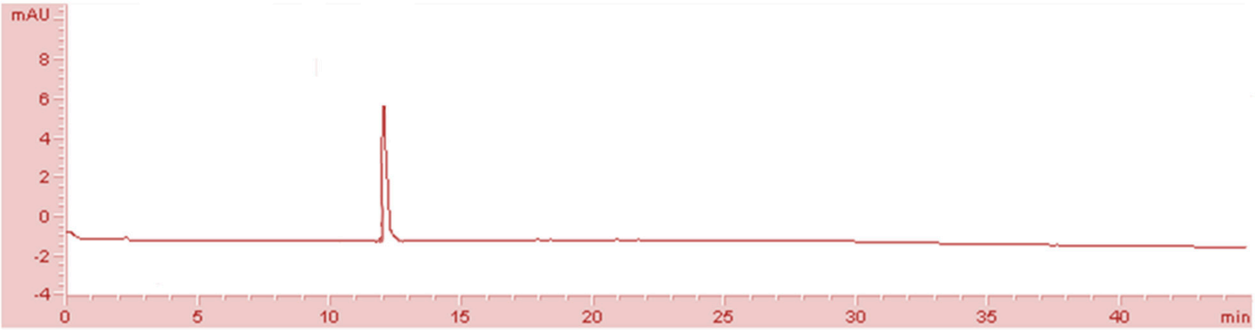

Supplement: Supplementary file 1 [file toxics-11-00698-s001.zip › toxics-2550284-supplementary.pdf]
